# Promoter RNA sequencing (PRSeq) for the massive and quantitative promoter analysis *in vitro*

**DOI:** 10.1038/s41598-019-39892-x

**Published:** 2019-02-28

**Authors:** Shoji Ohuchi, Thorsten Mascher, Beatrix Suess

**Affiliations:** 1 Institute of Microbiology, Technische Universität Dresden, Zellescher Weg 20b, 01217 Dresden, Germany; 2 Department of Biology, Technische Universität Darmstadt, Schnittspahnstrasse 10, 64287 Darmstadt, Germany; 3 Present Address: iBody Inc., Nagoya University Incubation Facility, Furo-cho 1, Chikusa-ku, Nagoya, 464-0814 Japan

## Abstract

Analysis of promoter strength and specificity is important for understanding and engineering gene regulation. Here, we report an *in vitro* promoter analysis method that can achieve both massiveness and quantitativeness. In this approach, a pool of single-stranded DNA with a partially randomized promoter sequence to be analyzed is chemically synthesized. Through enzymatic reactions, the randomized sequence will be copied to the downstream region, resulting in a template DNA pool that carries its own promoter information on its transcribed region. After *in vitro* transcription of the DNA pool with an RNA polymerase of interest, the sequences of the resulting transcripts will be analyzed. Since the promoter strength linearly correlates to the copy number of transcript, the strength of each promoter sequence can be evaluated. A model experiment of T7 promoter variants demonstrated the quantitativeness of the method, and the method was applied for the analysis of the promoter of cyanophage Syn5 RNA polymerase. This method provides a powerful approach for analyzing the complexity of promoter specificity and discrimination for highly abundant and often redundant alternative sigma factors such as the extracellular function (ECF) sigma factors.

## Introduction

Transcription initiation is the key step for controlling gene expression especially in bacterial and archaeal cells. Thus, analysis of promoter strength and specificity is important for understanding gene regulation. Traditionally, promoter analysis is performed employing *in vivo* reporter gene fusions [reviewed in^1^]. In these methods, promoter sequences of interest are fused to the coding sequence of a reporter protein, and the promoter activity is evaluated on the basis of the reporter protein expression. Because of the limitation of throughput, extensive analysis of related and mutant promoters is difficult to achieve by such methods, however.

Alternative methods employing *in vitro* transcription (IVTX) were developed to achieve higher throughput of promoter analysis^2–6^, [and reviewed in^1^]. Some of these *in vitro* methods easily covers more than 10^10^ sequence variations and thereby overcome the limited massiveness of *in vivo* methods. In addition, IVTX can eliminate the possible side effects originating from other endogenous RNA polymerases (RNAPs). *In vitro* methods therefore allow generating data free from false positive and negative signals. However, most available *in vitro* methods are not very quantitative. In addition, they have limitations on transcription conditions because IVTX should be coupled with another reaction for linking promoter sequence and its activity^1^.

To achieve both throughput and quantitativeness on promoter analysis, Nickels and colleagues recently developed a promoter analysis method employing a pool of template DNAs tagged with barcode sequence^7^. This method was successfully applied for analyzing effects of sequences of the transcription start site (TSS), the core recognition element, and the discriminator on TSS selection, transcriptional slippage, transcript yields, non-canonical capping^7–10^. While this method overcomes the limitations of previous *in vitro* methods, it requires large scale sequencing efforts for deconvoluting the barcode, which is very cost intensive.

Here, we have developed an alternative *in vitro* method employing a template DNA pool that carries its own promoter sequence information on the corresponding transcribed region. Because the promoter strength linearly correlates with the copy number of transcript, the strength of each promoter sequence can be evaluated by RNA sequencing. A model experiment of T7 promoter variants demonstrated the quantitativeness of the method. The method was also applied for the analysis of a promoter for cyanophage Syn5 RNAP. This method, termed “PRSeq (Promoter RNA Sequencing)”, will be applicable for extensive analyses and engineering of promoters.

## Materials and Methods

### DNA pool preparation

Template DNA pools were constructed by stepwise enzymatic reactions. The sequences of synthetic DNAs and all expected intermediate products during the pool preparations are shown in Supplemental Figs S1 and S2. All synthetic DNAs were purchased from Sigma-Aldrich Chemie (Darmstadt, Germany).

Initially, 400 pmol of synthetic DNAs including partially randomized phage promoters (NAI-Pt7-N6 and NAI-Ps5-N6 for T7 and Syn5 promoter variants, respectively) were subjected to fill-in elongation reaction. The reaction was performed with homemade recombinant *Taq* polymerase in 400-µL reaction volume by the following thermal conditions; 96 °C, 3 min; 50 °C, 2.5 min; 60 °C, 2.5 min; 72 °C, 5 min. The DNA product was recovered by phenol/chloroform extraction and ethanol precipitation.

Next, nicking and strand displacement elongation reactions in 200-µL volume were performed with Nt.Alw I (New England Biolabs, Ipswich, MA, USA) and Bst 2.0 DNA polymerase (New England Biolabs), respectively, according to the manufacture’s protocols. The DNA was recovered by phenol/chloroform extraction and ethanol precipitation.

For preventing undesired self-ligation in the following step, a single adenosine was added at the 3′-end to generate a cohesive end. The adenosine addition was performed with *Taq* polymerase in 200-µL volume at 68 °C for 10 min. The DNA sample was then fractionated by 8%-polyacrylamide gel electrophoresis (PAGE), and the corresponding DNA band was cut out from the gel. The gel slice was chopped into fine pieces and soaked in MilliQ water at room temperature overnight, followed by DNA recovery by ethanol precipitation.

To attach a primer-binding region for reverse transcription, a double-stranded linker DNA (NAI-lnkr-F and NAI-lnkr-R) was ligated to the recovered DNA with T4 DNA ligase (New England Biolabs) at 16 °C for 3 h. The ligated DNA was recovered by phenol/chloroform extraction and ethanol precipitation. To make fully double-stranded DNA, the ligated DNA was subjected to fill-in elongation reaction with *Taq* polymerase at 60 °C for 10 min. The DNA sample was fractionated by 6%-PAGE, and the ligated DNA was recovered as described above. The DNA was solved in 40-µL MilliQ water and stored at −20 °C until use. The qualities of the starting pools were analyzed by sequencing.

For the control reaction of Syn5 RNAP, the control DNA pool was prepared by PCR with *Taq* polymerase employing the test DNA pool prepared above, NAIs5-Fpc (5′-GGTGGAGGCG GATCGATTGG GCACCCGTAA GAGGTTTTCC-3′), and NAI-lnkr-R as a template DNA, forward primer, and reverse primer, respectively. The amplified fragment carries wild-type Syn5 promoter sequence and the copy of randomized promoter sequence on the transcribed region of the template pool DNA. The DNA was purified and stored as described above.

### *In vitro* transcription (IVTX) and sequence analysis

Recombinant T7 and Syn5 RNA polymerases (RNAPs) were prepared as previously described^11,12^. For PRSeq analysis, 30-µL *in vitro* transcription (IVTX) was performed in 40 mM Tris-HCl (pH 8.0), 6 mM MgCl_2_, 10 mM dithiothreitol (DTT), 2 mM spermidine, 0.25 mM each rNTPs, 20 U RNase inhibitor (MoloX, Berlin, Germany), RNAP (50 nM T7 RNAP or 300 nM Syn5 RNAP), and 500 nM of the above prepared template DNA at 37 °C (for T7 RNAP) or at 30 °C (for Syn5 RNAP) for 30 min. After the IVTX, the template DNA was digested with 2 U DNase I (New England Biolabs) at 37 °C for 15 min. The sample was passed through MicroSpin G-25 columns (GE Healthcare Bio-Sciences, Pittsburgh, PA, USA), and the RNA was recovered by phenol/chloroform extraction and ethanol precipitation.

For cloning of the pool variants, the recovered RNA was reverse transcribed with SuperScript II reverse transcriptase (Thermo Fisher Scientific, Waltham, MA, USA) employing the primer NAI-lnkr-R. The resulting cDNA was then amplified by PCR with *Taq* DNA polymerase employing primers NAI-lnkr-R and NAI-s5-F. The amplified DNA was cloned by CloneJET PCR Cloning Kit (Thermo Fisher Scientific) and transformed into *E*. *coli* 10-beta cells (New England Biolabs). The transformants were streaked onto LB-agar plates containing ampicillin and grown at 37 °C overnight. Plasmid preparation and sequencing of each clones were performed at Seqlab GmbH (Göttingen, Germany).

The sequence frequency (F_n, b_) of each point mutation of T7 promoter was calculated from the reported promoter activities (P_n, b_) of single point mutations^13^ based on the following equation, where n and b indicate each residue position randomized and residue base (A, C, G, or T), respectively.

$${{\rm{F}}}_{{\rm{n}},{\rm{b}}}={{\rm{P}}}_{{\rm{n}},{\rm{b}}}/{(P}_{{\rm{n}},{\rm{A}}}+{{\rm{P}}}_{{\rm{n}},{\rm{C}}}+{{\rm{P}}}_{{\rm{n}},{\rm{G}}}+{{\rm{P}}}_{{\rm{n}},{\rm{T}}})$$

The promoter activity of each point mutation of T7 or Syn5 promoter was estimated from the sequence frequencies of the promoter pool after PRSeq reaction based on the following equation, where P_wt_ and F_wt_ indicate the promoter activity and sequence frequency of the promoter without mutations.

$${{\rm{P}}}_{{\rm{n}},{\rm{b}}}={{\rm{P}}}_{{\rm{wt}}}\times {{\rm{F}}}_{{\rm{n}},{\rm{b}}}/{{\rm{F}}}_{{\rm{wt}}}$$

For promoter activity assay of the Syn5 promoter variants, 10-µL IVTX was performed in 40 mM Tris-HCl (pH 8.0), 6 mM MgCl_2_, 10 mM DTT, 2 mM spermidine, 100 nM DNA, 0.25 mM each rNTPs, [alpha-^32^P]-UTP [Hartmann Analytic (Braunschweig, Germany) or PerkinElmer (Waltham, MA, USA)], 300 nM Syn5 RNAP, and 4 U RNase inhibitor at 30 °C for 30 min. All the sequences of template DNAs were shown in Supplemental Table S1. The reaction was stopped by adding 10-µL Stop Solution [90% formamide, 20 mM ethylenediaminetetraacetic acid (pH 8.0), and 0.005% xylene cyanol]. The transcript was separated by 8%-denaturing PAGE containing 8 M urea, and band images were analyzed by FLA-5000 (Fujifilm, Tokyo, Japan) or by Typhoon Trio (GE Healthcare Bio-Sciences).

## Results

### Design of PRSeq (Promoter RNA Sequencing) method

**Figure 1:**
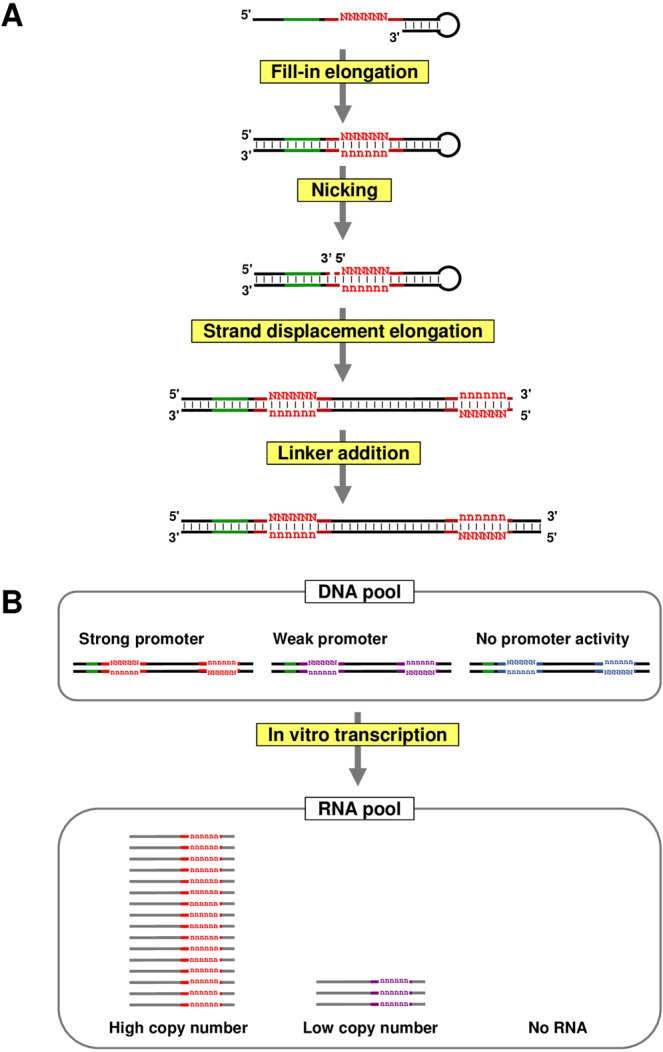
Schematic diagram of PRSeq (Promoter RNA Sequencing) method. (**A**) DNA pool preparation. Synthetic single-stranded DNA with a 3′-terminal hairpin structure is subjected to intra-strand fill-in DNA elongation to generate double-stranded recognition sequence for nicking enzyme, Nt.AlwI (green). The randomized promoter region is indicated by red color. After the nicking reaction, the DNA is subjected to DNA elongation employing Bst 2.0 DNA polymerase which has strong strand displacement activity. In this reaction, the 5′-DNA fragment generated by the nicking acts as a primer, and the random sequence in the promoter region is copied to downstream of the promoter. Finally, a primer sequence for reverse transcription is attached by ligation. Note that a part of non-randomized region of the promoter is not copied, and the copied sequence should not act as the promoter. (**B**) *In vitro* transcription (IVTX) of the DNA pool. The DNA pool prepared by the reactions in (**A**) is applied for IVTX. The template DNA molecules with strong and weak promoters (shown by red and purple colors) generate high and low copies of RNA, respectively, whereas most of the DNA with non-functional promoter variant (shown by blue color) do not. Since the RNA copy number is in proportion to the promoter activity linearly, activity of each promoter sequence can be evaluated by RNA sequencing of the transcript pool.

Recent progress of sequencing technology enables massive analyses of RNA population, and RNA sequencing has been applied for analyzing population enrichment during *in vitro* RNA selection and for analyzing residue properties of structural RNAs^14–17^. However, this technology cannot be applied for *in vitro* promoter analysis because the promoter sequence is not a part of the transcribed region, and hence, the resulting transcripts loose the information of their own promoter. For maintaining promoter sequence information inside the resulting transcript, we developed a preparation procedure for generating template DNA pools, during which the promoter sequence is copied onto the downstream transcribed region (Fig. 1A).

The procedure consists of four reaction steps. In the first step, synthetic single-stranded DNA with a terminal hairpin structure is subjected to intra-strand fill-in DNA elongation. The synthetic DNA carries a single-stranded region that contains (i) the recognition sequence for a nicking enzyme and (ii) the partially randomized promoter sequence to be analyzed. The elongation reaction generates a double-stranded DNA fragment of these regions. Next, an upstream position of the random sequence is nicked by the nicking enzyme. In the third step, this nicked DNA is subjected to DNA elongation employing a DNA polymerase which has strong strand displacement activity. In this reaction, the 5′-DNA fragment generated by the nicking acts as a primer, and the random sequence in the promoter region is copied to a fragment downstream of the promoter. Finally, a linker sequence for reverse transcription is attached downstream of the copied random sequence.

*In vitro* transcription (IVTX) by an RNA polymerase (RNAP) of interest is then performed employing this DNA pool as a template (Fig. 1B). It is expected that the copy number of a transcript generated from a template DNA is proportional to the relative promoter activity on the template DNA. Thus, the promoter activity of each promoter sequence should correlate directly with the copy numbers of each promoter sequence in the transcribed RNA pool.

### Evaluation of T7 promoter sequence

To validate the designed PRSeq method, we performed a model experiment employing T7 RNAP. We designed a DNA pool of partially randomized T7 promoter. In this pool, positions -12 to -7, the residues determining the RNAP specificity^18^, were randomized (Supplemental Fig. S1). As judged from the band intensities on PAGE, each reaction steps except for the ligation proceeded more than 90% efficiency. In contrast, the ligation proceeded less efficiently (~60%) probably due to the loss of the 5′-phosphate group and incompleteness of the 3′-adenosine addition. The pool quality was estimated by sequencing the cloned pool DNAs, and we confirmed the quality satisfaction, that is, unbiased distribution of nucleotides over the randomized residues and complete copies of the random sequences onto the transcribed regions (Supplemental Table S2).

**Figure 2:**
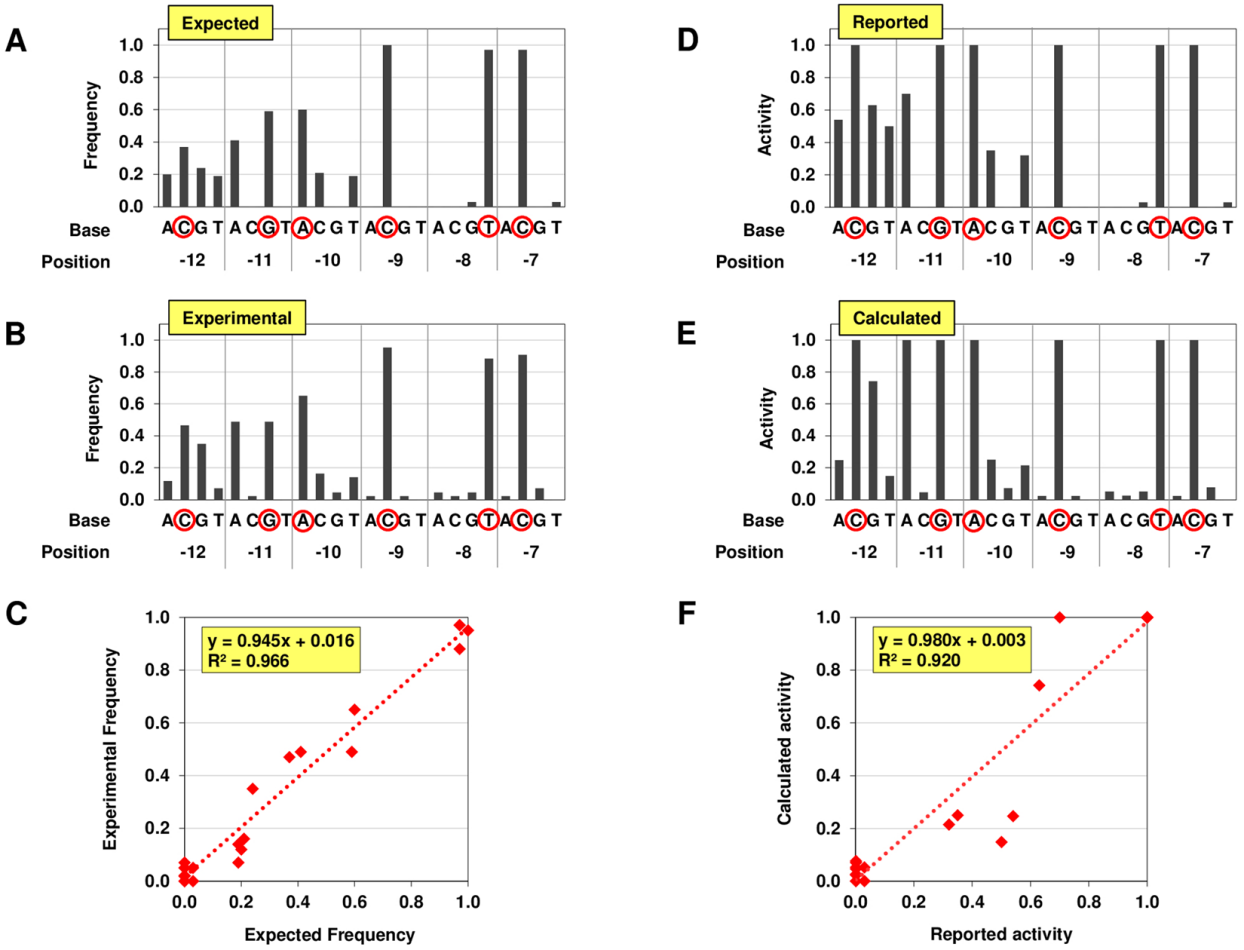
Model experiment employing T7 RNA polymerase (RNAP). (**A**) Expected sequence frequency of randomized -12 to -7 region of T7 promoter in the enriched pool. The frequency is calculated according to the promoter activities of single mutations reported previously^13^. Note that the reported activities as ‘less than 0.03’ were treated as ‘0’ for the calculation. (**B**) Experimentally obtained sequence frequency in the enriched pool. After the enrichment for T7 promoter activity employing the DNA pool with randomized -12 to -7 region of T7 promoter, variants in the pool was cloned, and 43 clones were randomly picked out for sequencing. The sequences of each clones are shown in Supplemental Table S2. (**C**) Comparison of the expected and experimentally obtained sequence frequencies. The frequencies of each nucleotide of each position of the data of (**A**,**B**) are plotted. (**D**) Experimentally determined promoter activities of single mutations of T7 promoter. The data is from the previous study^13^. The reported activities as ‘less than 0.03’ are shown as ‘0’ in the graph. (**E**) Promoter activities of single mutations of T7 promoter determined by PRSeq. The activities are calculated from the sequence frequency in (**F**) Comparison of the experimentally determined and PRSeq-estimated promoter activities. The promoter activities of single mutations of the data of (**D**,**E**) are plotted. The wild-type promoter sequence is emphasized by red circles.

This template DNA pool was subjected to IVTX with T7 RNAP, and PRSeq reaction was carried out. For the analysis, sequences of 43 clones from the enriched pool were determined (Supplemental Table S3). Because the precise promoter activities of single point mutations within the randomized residues were reported previously^13^, the sequence frequency in the enriched pool can be predicted (Fig. 2A). We found that the experimentally obtained sequence frequency of the enriched pool is highly consistent with the expected frequency (Fig. 2B). When the frequencies of each mutations are plotted, the data showed a strong correlation between the expected and experimental frequencies [the coefficient of determination (R^2^) value of 0.966 (Fig. 2C)].

The promoter activities of the variants were calculated from the obtained sequence frequency. As expected, the overall profile of the calculated activities was similar to that of the reported activities (Fig. 2D,E), and the data showed a fine correlation with the R^2^ value of 0.920 (Fig. 2F). This result demonstrates appropriate design of the method and applicability for the quantitative promoter analysis.

### Analysis of Syn5 RNAP promoter

Next, we employed PRSeq method to analyze the promoter of the cyanophage Syn5 RNAP, a single-subunit RNAP homologous to T7 RNAP^19^. In contrast to T7 RNAP, Syn5 RNAP efficiently accepts not only adenosine and guanine but also cytosine as the initializing nucleotide for transcription^20^. In addition, Syn5 RNAP generates the precise run-off transcripts with homogeneous 3′-termini^20^. These unique features suggest the usefulness of the RNAP for IVTX, and the promoter analysis would be valuable for a wide range of applications.

For the analysis, we prepared a template DNA pool in which positions -11 to -6 of the known Syn5 promoter (hereafter, the wild-type promoter) were randomized (Supplemental Fig. S2). In contrast to the DNA pool of the randomized T7 promoter, sequencing analysis of the cloned pool DNAs indicated biased nucleotide composition in the randomized region; adenosine content through positions -11 to -8 was 7.3% whereas 25% was desired (Supplemental Table S4). This bias was probably due to a non-uniform incorporation of each nucleotide during the chemical DNA synthesis. Because such a bias of the starting pool could make data interpretation difficult, we also prepared a control DNA pool in which the randomized promoter was replaced with the wild-type promoter, whereas the copy of promoter sequence on the transcribed region remained randomized. This control DNA pool was subjected to PRSeq reaction in parallel, and applied for adjusting the sequence bias of the test DNA pool.

**Figure 3:**
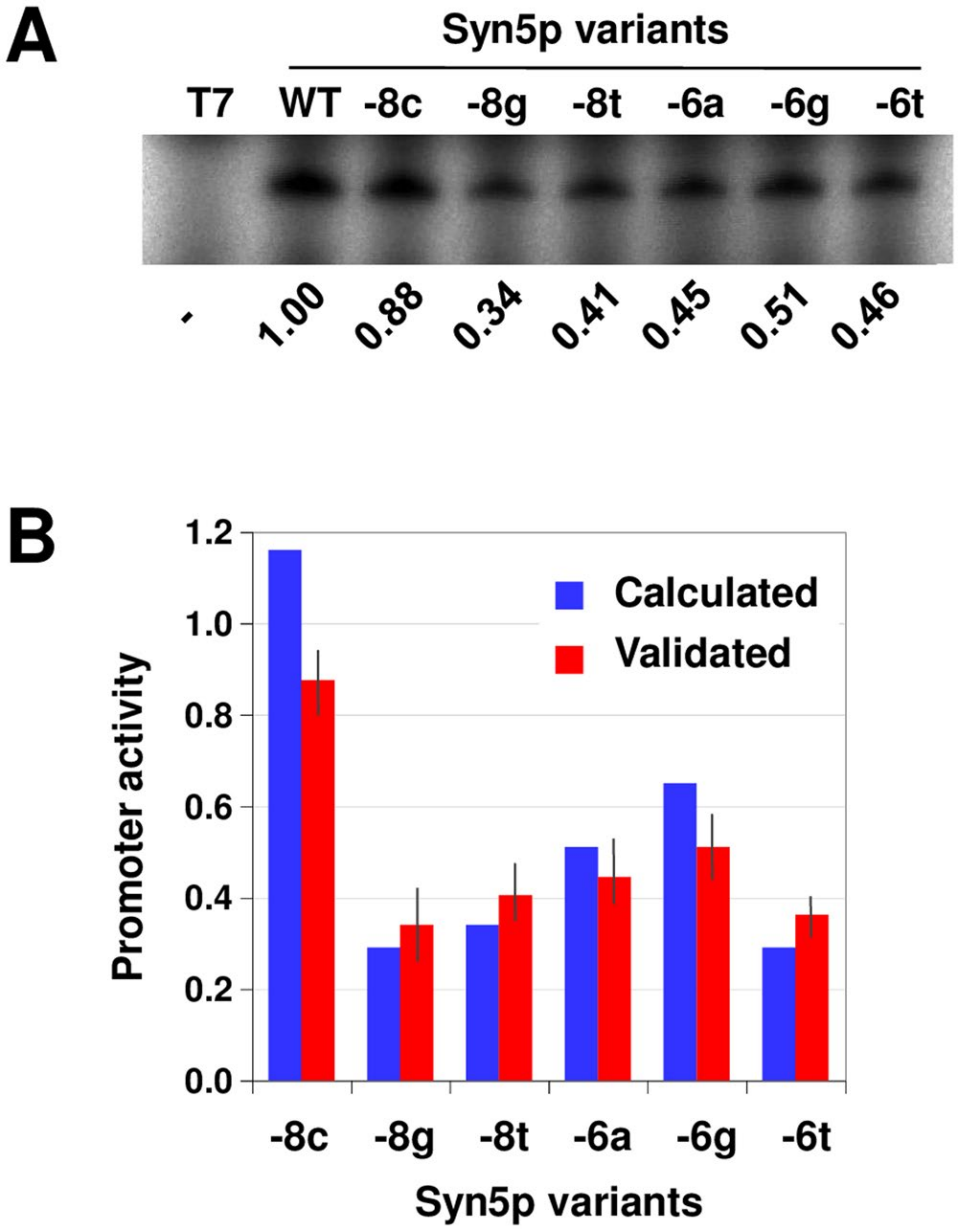
Analysis of Syn5 promoter sequence. (**A**) Validation of promoter activities of Syn5 promoter variants. Template DNAs with six single mutations of Syn5 promoter were subjected for IVTX. As a negative control and positive standard, the reactions employing the DNAs with T7 promoter (T7) and wild-type Syn5 promoter (WT), respectively, were also carried out. Mean values of three independent duplicate experiments were shown under the gel image. Mutations A(-8)C, A(-8)G, A(-8)T, C(-6)A, C(-6)G, and C(-6)T are indicated as -8c, -8g, -8t, -6a, -6g, and -6t, respectively. (**B**) Comparison of the experimentally determined and PRSeq-estimated promoter activities. The data of the indicated single mutations of Syn5 promoter in (**A**) and Supplemental Fig. S3 are shown. Error bars indicate standard deviations.

After IVTX, reverse transcription-PCR, and cloning, 57 and 50 colonies from the control pool and test pool, respectively, were randomly picked out for sequencing (Supplemental Tables S5 and S6). As expected, sequence frequency of the enriched test pool showed apparent difference from frequency of the control pool, and sequences similar to the wild-type Syn5 promoter were enriched (Supplemental Fig. S3A,B). According to the frequencies of these pools, the promoter activities of single mutations across positions -11 to -6 of Syn5 promoter were calculated (Supplemental Fig. S3C). To validate the calculated activities, promoter activities of single mutations on positions -8 and -6 were experimentally determined (Fig. 3A). According to PRSeq result, variant -8c would retain the activity comparable to the wild-type promoter, and other variants would moderately loose the activity (Fig. 3B and Supplemental Fig. S3C). As intended, the overall tendency of these calculated activities was similar to that of the data experimentally obtained (Fig. 3B).

## Discussion

Detailed promoter analyses are important for understanding the mechanism of gene regulation that is mainly achieved during transcription initiation in bacteria and archaea. While *in vivo* methods are widely applied for promoter analyses, the limited massiveness prevents extensive analyses of promoter sequences^1^. Towards that goal, *in vitro* methods are advantageous while they have limitations in this regard to quantitativeness and transcription conditions. In this study, we developed a massive and quantitative method, PRSeq, for the analysis of promoter specificity and strength *in vitro*. Because IVTX and other reactions are uncoupled in PRSeq method, this method accepts any desired transcription conditions.

The recently developed promoter analysis method employing a pool of template DNAs tagged with barcode sequence also overcomes the limitations of previous *in vitro* methods^7^. However, the barcode deconvolution for this method requires complete sequencing of a whole set of pool DNAs, since the linkage between the barcode and the promoter sequences cannot be predicted *a priori*. In contrast, the PRSeq method generates a DNA pool that carries its own promoter information on its transcribed region. Thus, only a limited set of the pool DNAs needs to be sequenced for evaluating the sequence bias in our method. For example, PRSeq method required less than 100 sequence data at least for preliminary validation of sequence preferences over six residues in our case. We speculate a single reaction of next generation sequencing would be sufficient for the analyses of multiple promoters.

Thus, PRSeq provides a very powerful approach for analyzing the complexity of promoter recognition and particularly discrimination for organisms with large sets of alternative sigma factors. The extracytoplasmic function (ECF) are the most widely distributed and diverse group of alternative sigma factors^21–23^. They can be particularly enriched in the genomes of complex microorganisms, such as the actinobacteria sometimes encoding over 100 ECF sigma factors^24^. This complexity is further enhanced by the redundancy of highly homologous ECF sigma factors belonging to the same ECF subgroup, which – in theory – should recognize highly similar alternative promoters^25^. Because of this challenge, so far only very few time-consuming studies have comprehensively addressed the question of promoter discrimination between highly similar ECF sigma factors in a given organism, most notably for ECF-model bacterium *Bacillus subtilis*^26,27^. Here, PRSeq now offers a very direct and efficient means to determine the regulatory overlap or promoter discrimination at very closely related promoters from paralogous ECF sigma factors of the same ECF subgroup. Combining the PRSeq method developed in this study, with high-throughput reconstitution of RNAPs employing cell-free protein synthesis^28^, will be even more powerful in allowing multiplex analyses of promoter specificities for sets of these sigma factors.

## Supplementary information

Supplementary Information
