# Supplementary material for: Promoter RNA sequencing (PRSeq) for the massive and quantitative promoter analysis in vitro
Source: Sci Rep. 2019 Feb 28;9:3118. doi: 10.1038/s41598-019-39892-x (PMC6395800; doi:10.1038/s41598-019-39892-x)
Supplement: Supplementary file 1 — Supplementary Information [file 41598_2019_39892_MOESM1_ESM.pdf]

Supplementary Information

Promoter RNA sequencing (PRSeq) for the massive and quantitative promoter analysis *in vitro*

Shoji OHUCHI[1, a, b], Thorsten MASCHER[1], and Beatrix SUESS[2]

[1]Institute of Microbiology, Technische Universität Dresden, Zellescher Weg 20b, 01217 Dresden, Germany

[2]Department of Biology, Technische Universität Darmstadt, Schnittspahnstrasse 10, 64287 Darmstadt, Germany

Contents

Supplemental Figure S1. DNA pool construction for the analysis of T7 promoter.

Supplemental Figure S2. Supplemental Figure S2. DNA pool construction for the analysis of Syn5 promoter.

Supplemental Figure S3. Syn5 promoter analysis by PRSeq.

Supplemental Figure S4. Supplemental Figure S4. Full-length gel image for the validation of promoter activities of Syn5 promoter variants.

Supplemental Table S1. Sequences of template DNAs for the evaluation of mutations on Syn5 promoter.

Supplemental Table S2. Sequences of clones from template DNA pool for T7 promoter analysis.

Supplemental Table S3. Sequences of clones from the enriched T7 promoter pool.

Supplemental Table S4. Sequences of clones from template DNA pool for Syn5 promoter analysis.

Supplemental Table S5. Sequences of clones from the control Syn5 promoter pool.

Supplemental Table S6. Sequences of clones from the enriched Syn5 promoter pool.

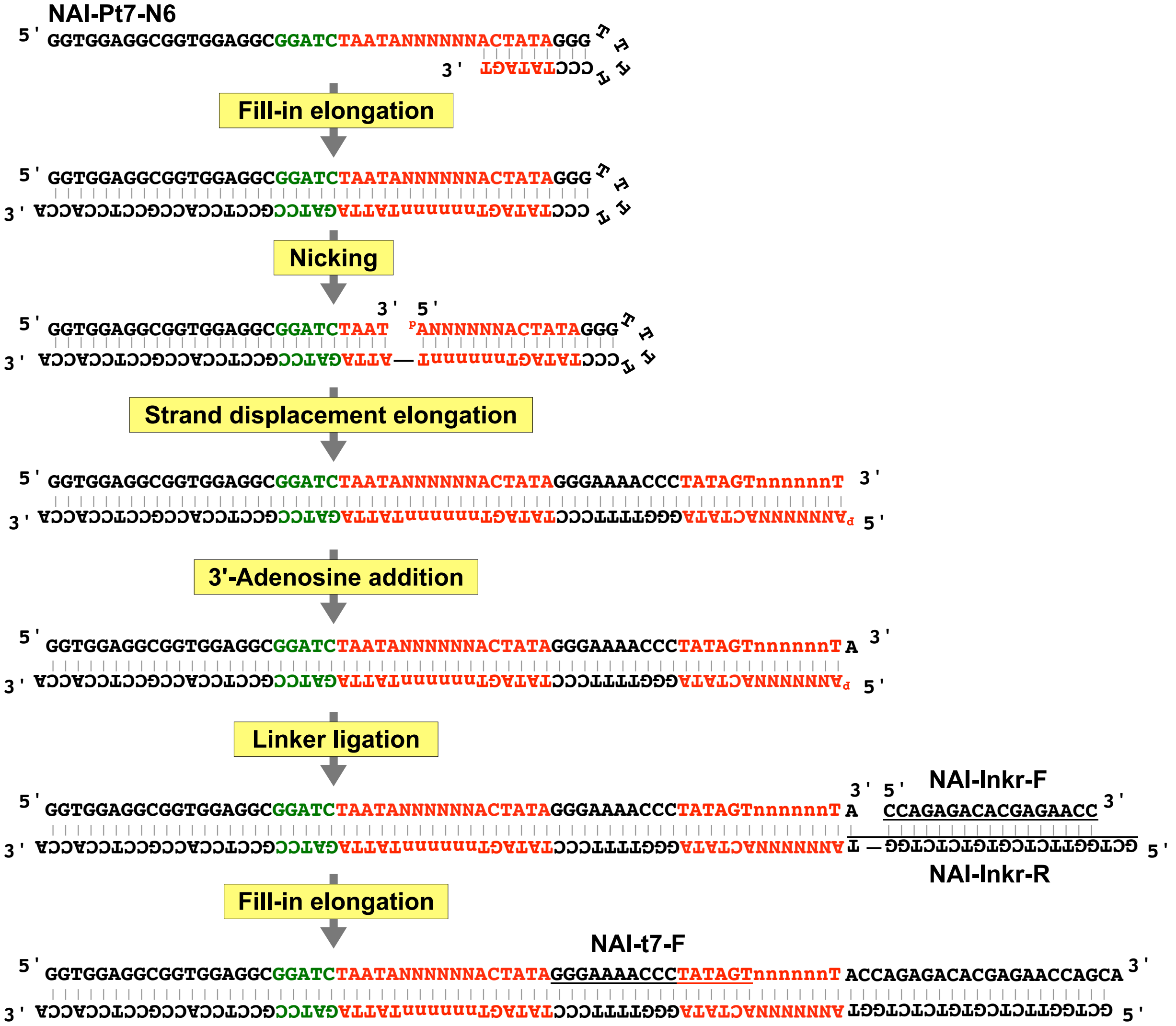

Supplemental Figure S1. DNA pool construction for the analysis of T7 promoter. The DNA pool was prepared by the following reaction steps: 1) Fill-in elongation by Taq polymerase; 2) Nicking reaction by Nt.Alw I; 3) Strand replacement elongation by Bst 2.0; 4) 3'-adenosine addition by Taq polymerase; 5) Linker ligation by T4 DNA ligase; 6) Fill-in elongation by Taq DNA polymerase. Through the serial reactions, the randomized promoter sequence is copied onto the transcribed region. Partially randomized T7 promoter sequence and the Nt.Alw I recognition site are shown by red and green letters, respectively. Primer binding regions are emphasized by underlines. Superscript "P" indicates 5'-phosphate group generated by the nicking reaction and required for the ligation reaction.

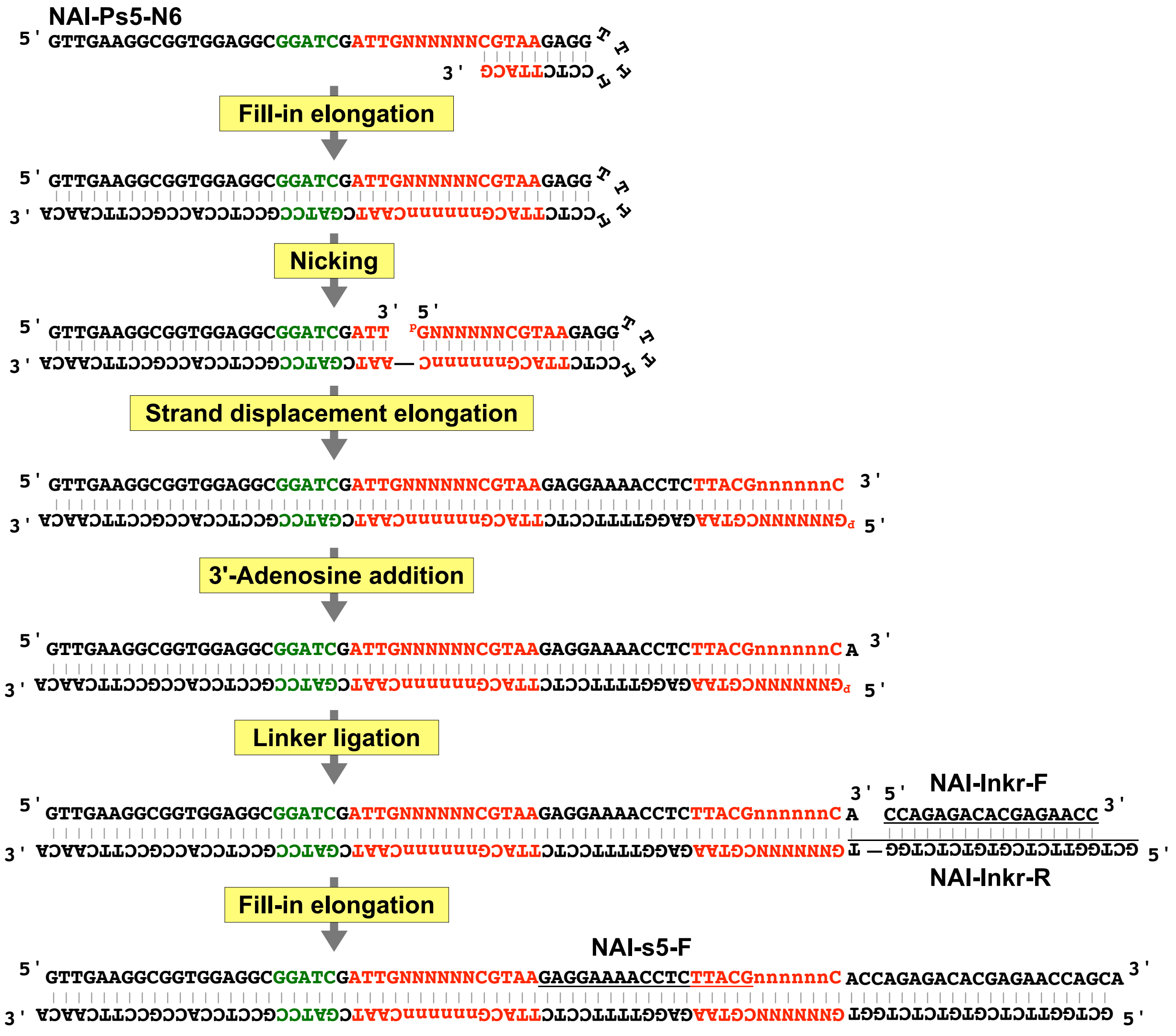

Supplemental Figure S2. DNA pool construction for the analysis of Syn5 promoter.
For the Figure explanation, see Supplemental Figure S1.

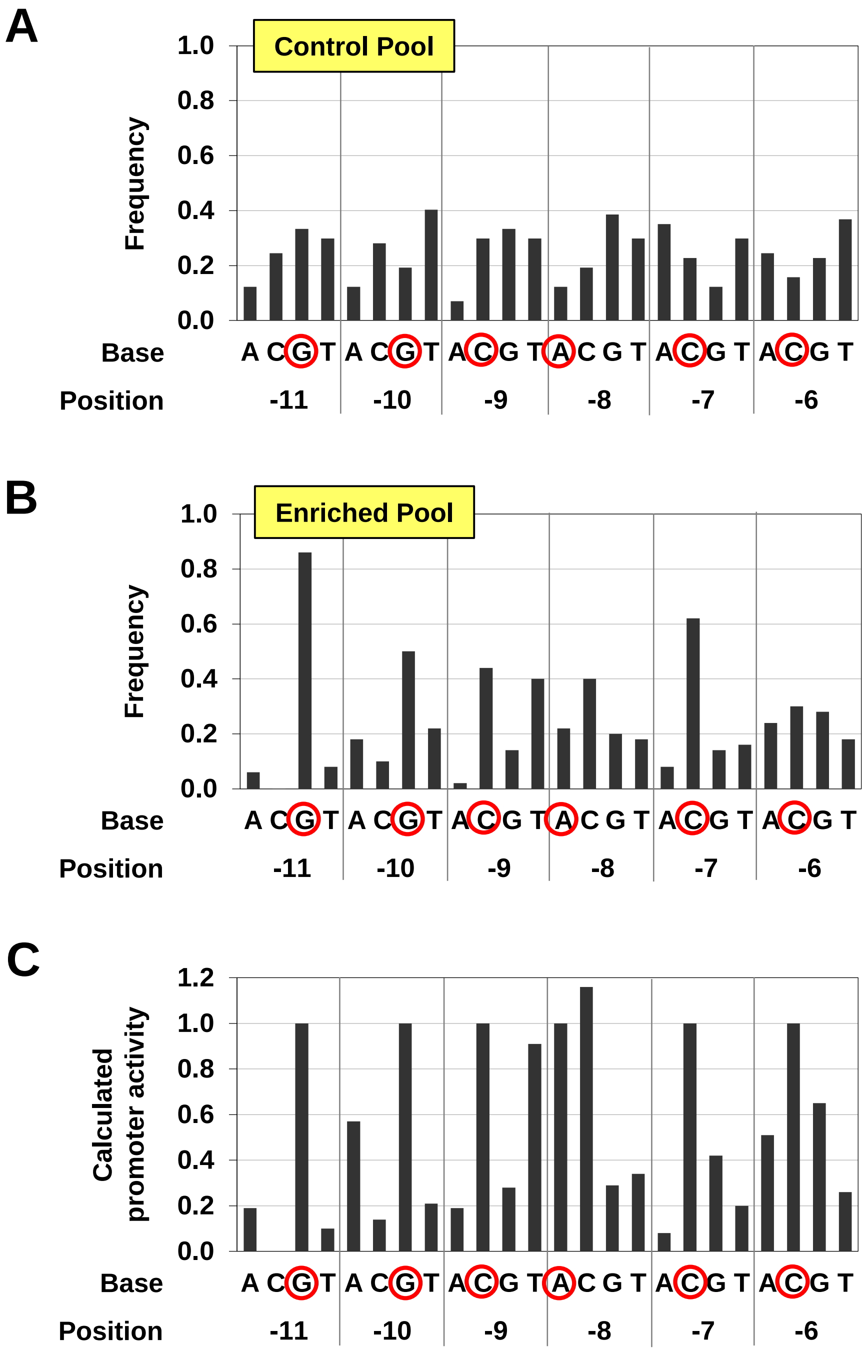

Supplemental Figure S3. Syn5 promoter analysis by PRSeq. (A) Sequence frequency of randomized -11 to -6 region of Syn5 promoter in the control pool. After the control reaction, variants in the pool were cloned, and 57 clones were randomly picked out for sequencing. The wild-type promoter sequence is emphasized by red circles. The clone sequences are listed in Supplemental Table S2. **(B)** Sequence frequency of randomized -11 to -6 region of Syn5 promoter in the enriched pool. After the PRSeq employing Syn5 RNAP, variants in the pool were cloned, and 50 clones were randomly picked out for sequencing. The clone sequences are listed in Supplemental Table S3. **(C)** Promoter activities of Syn5 promoter variants calculated according to the sequence frequencies of above figures.

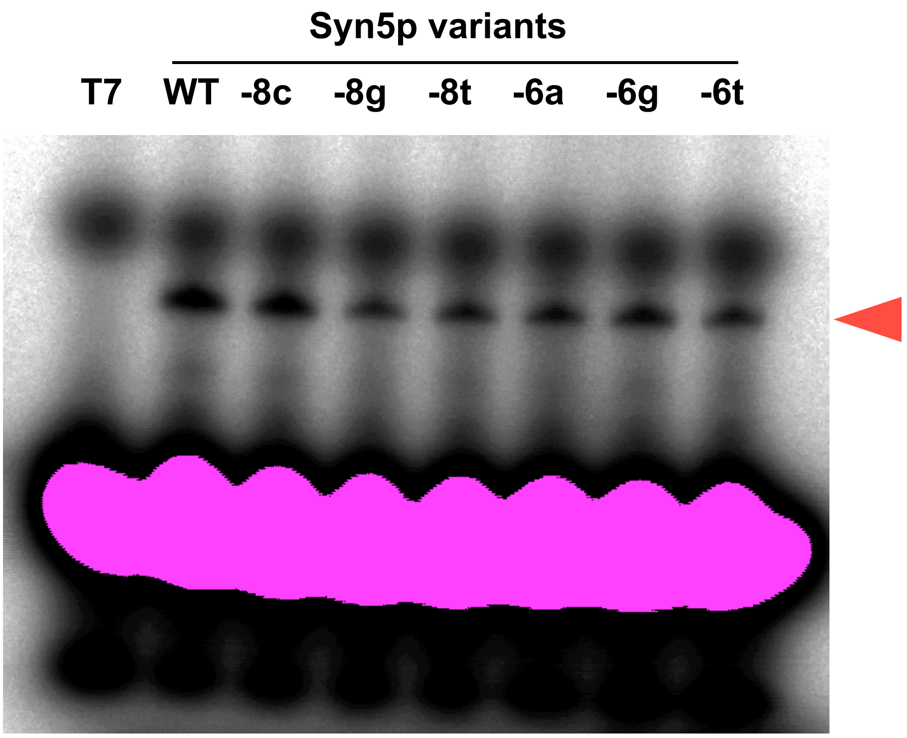

Supplemental Figure S4. Full-length gel image for the validation of promoter activities of Syn5 promoter variants.
The full-length image of the gel displayed in Figure 3A is shown. Template DNAs with six single mutations of Syn5 promoter were subjected for IVTX. As a negative control and positive standard, the reactions employing the DNAs with T7 promoter (T7) and wild-type Syn5 promoter (WT), respectively, were also carried out. Mean values of three independent duplicate experiments were shown under the gel image. Mutations A(-8)C, A(-8)G, A(-8)T, C(-6)A, C(-6)G, and C(-6)T are indicated as -8c, -8g, -8t, -6a, -6g, and -6t, respectively. The position of the corresponding transcripts is indicated by red arrowhead.

Supplemental Table S1. Sequences of template DNAs for the evaluation of mutations on Syn5 promoter

Name	Sequence (5'-to-3')[a]	Promoter
T7p-D01	TAATACGACT CACTATAGGG ATCGACAGGG CTCCCGATTG GCTCGGCGAA<br>GATCGCCTGT ACTGTGGAAA GCCGAGTCGT G	T7 promoter (negative control)
Syn5p-D01	GGTATTGGGC ACCCGTAAGG GATCGACAGG GCTCCCGATT GGCTCGGCGA<br>AGATCGCCTG TACTGTGGAA AGCCGAGTCG TG	Wild-type Syn5 promoter
Syn5p(-8c)-D01	GGTATTGGGC cCCCGTAAGG GATCGACAGG GCTCCCGATT GGCTCGGCGA<br>AGATCGCCTG TACTGTGGAA AGCCGAGTCG TG	A(-8)C mutant of Syn5 promoter
Syn5p(-8g)-D01	GGTATTGGGC gCCCGTAAGG GATCGACAGG GCTCCCGATT GGCTCGGCGA<br>AGATCGCCTG TACTGTGGAA AGCCGAGTCG TG	A(-8)G mutant of Syn5 promoter
Syn5p(-8t)-D01	GGTATTGGGC tCCCGTAAGG GATCGACAGG GCTCCCGATT GGCTCGGCGA<br>AGATCGCCTG TACTGTGGAA AGCCGAGTCG TG	A(-8)T mutant of Syn5 promoter
Syn5p(-6a)-D01	GGTATTGGGC ACaCGTAAGG GATCGACAGG GCTCCCGATT GGCTCGGCGA<br>AGATCGCCTG TACTGTGGAA AGCCGAGTCG TG	C(-6)A mutant of Syn5 promoter
Syn5p(-6g)-D01	GGTATTGGGC ACgCGTAAGG GATCGACAGG GCTCCCGATT GGCTCGGCGA<br>AGATCGCCTG TACTGTGGAA AGCCGAGTCG TG	C(-6)G mutant of Syn5 promoter
Syn5p(-6t)-D01	GGTATTGGGC ACtCGTAAGG GATCGACAGG GCTCCCGATT GGCTCGGCGA<br>AGATCGCCTG TACTGTGGAA AGCCGAGTCG TG	C(-6)T mutant of Syn5 promoter

a) Promoter sequences are emphasized by underlines, and mutations are indicated by lowercase letters.

Supplemental Table S2. Sequences of clones from template DNA pool for T7 promoter analysis[a]

GGTGGAGGCGGTGGAGGCGGATCTAATA TTTCGA ACTATA*GGGAAAACCCTATAGT TCGAAA TACCACGATCCCGCTCCAGCTCA*
GGTGGAGGCGGTGGAGGCGGATCTAATA GGCATC ACTATA*GGGAAAACCCTATAGT GATGCC TACCACGATCCCGCTCCAGCTCA*
GGTGGAGGCGGTGGAGGCGGATCTAATA GTGAGT ACTATA*GGGAAAACCCTATAGT ACTCAC TACCACGATCCCGCTCCAGCTCA*
GGTGGAGGCGGTGGAGGCGGATCTAATA GACGTT ACTATA*GGGAAAACCCTATAGT AACGTC TACCACGATCCCGCTCCAGCTCA*
GGTGGAGGCGGTGGAGGCGGATCTAATA CTTGGT ACTATA*GGGAAAACCCTATAGT ACCAAG TACCACGATCCCGCTCCAGCTCA*
GGTGGAGGCGGTGGAGGCGGATCTAATA GTTTGG ACTATA*GGGAAAACCCTATAGT CCAAAC TACCACGATCCCGCTCCAGCTCA*
GGTGGAGGCGGTGGAGGCGGATCTAATA GACAGC ACTATA*GGGAAAACCCTATAGT GCTGTC TACCACGATCCCGCTCCAGCTCA*
GGTGGAGGCGGTGGAGGCGGATCTAATA GTCCGG ACTATA*GGGAAAACCCTATAGT CCGGAC TACCACGATCCCGCTCCAGCTCA*
GGTGGAGGCGGTGGAGGCGGATCTAATA AGGCTG ACTATA*GGGAAAACCCTATAGT CAGCCT TACCACGATCCCGCTCCAGCTCA*
GGTGGAGGCGGTGGAGGCGGATCTAATA AGTTGA ACTATA*GGGAAAACCCTATAGT TCAACT TACCACGATCCCGCTCCAGCTCA*
GGTGGAGGCGGTGGAGGCGGATCTAATA TTGAAT ACTATA*GGGAAAACCCTATAGT ATTCAA TACCACGATCCCGCTCCAGCTCA*
GGTGGAGGCGGTGGAGGCGGATCTAATA AAATGA ACTATA*GGGAAAACCCTATAGT TCATTT TACCACGATCCCGCTCCAGCTCA*
GGTGGAGGCGGTGGAGGCGGATCTAATA TTCGTT ACTATA*GGGAAAACCCTATAGT AACGAA TACCACGATCCCGCTCCAGCTCA*
GGTGGAGGCGGTGGAGGCGGATCTAATA CGTGTC ACTATA*GGGAAAACCCTATAGT GACACG TACCACGATCCCGCTCCAGCTCA*
GGTGGAGGCGGTGGAGGCGGATCTAATA CGGTGG ACTATA*GGGAAAACCCTATAGT CCACCG TACCACGATCCCGCTCCAGCTCA*
GGTGGAGGCGGTGGAGGCGGATCTAATA CTTCGG ACTATA*GGGAAAACCCTATAGT CCGAAG TACCACGATCCCGCTCCAGCTCA*
GGTGGAGGCGGTGGAGGCGGATCTAATA TTTCTT ACTATA*GGGAAAACCCTATAGT AAGAAA TACCACGATCCCGCTCCAGCTCA*
GGTGGAGGCGGTGGAGGCGGATCTAATA AGAGTG ACTATA*GGGAAAACCCTATAGT CACTCT TACCACGATCCCGCTCCAGCTCA*
GGTGGAGGCGGTGGAGGCGGATCTAATA GCTCCT ACTATA*GGGAAAACCCTATAGT AGGAGC TACCACGATCCCGCTCCAGCTCA*
GGTGGAGGCGGTGGAGGCGGATCTAATA ATTGAG ACTATA*GGGAAAACCCTATAGT CTCAAT TACCACGATCCCGCTCCAGCTCA*
GGTGGAGGCGGTGGAGGCGGATCTAATA TTTTTC ACTATA*GGGAAAACCCTATAGT GAAAAA TACCACGATCCCGCTCCAGCTCA*
GGTGGAGGCGGTGGAGGCGGATCTAATA GCATTT ACTATA*GGGAAAACCCTATAGT AAATGC TACCACGATCCCGCTCCAGCTCA*
GGTGGAGGCGGTGGAGGCGGATCTAATA GACGTG ACTATA*GGGAAAACCCTATAGT CACGTC TACCACGATCCCGCTCCAGCTCA*
GGTGGAGGCGGTGGAGGCGGATCTAATA GCGTGG ACTATA*GGGAAAACCCTATAGT CCACGC TACCACGATCCCGCTCCAGCTCA*
GGTGGAGGCGGTGGAGGCGGATCTAATA CTGGCG ACTATA*GGGAAAACCCTATAGT CGCCAG TACCACGATCCCGCTCCAGCTCA*
GGTGGAGGCGGTGGAGGCGGATCTAATA CTTTGA ACTATA*GGGAAAACCCTATAGT TCAAAG TACCACGATCCCGCTCCAGCTCA*
Wild-type T7 promoter: TAATA CGACTC ACTATA

a) The sequences are represented as 5'-to-3' orientation. The six nucleotides between spaces indicate the randomized region. The transcribed region is emphasized by Italics. For reference, the wild-type T7 promoter sequence is shown at the bottom of the table.

Supplemental Table S3. Sequences of clones from the enriched T7 promoter pool[a]

TGAGCTGGAGCGGGATCGTGGTA	GAGCTC	ACTATAGGGTTTTCCCTGTGAAGTGAA
TGAGCTGGAGCGGGATCGTGGTA	CGACGC	ACTATAGGGTTTTCCCTGTGAAGTGAA
TGAGCTGGAGCGGGATCGTGGTA	TGACTC	ACTATAGGGTTTTCCCTGTGAAGTGAA
TGAGCTGGAGCGGGATCGTGGTA	CGACTC	ACTATAGGGTTTTCCCTGTGAAGTGAA
TGAGCTGGAGCGGGATCGTGGTA	CAACTC	ACTATAGGGTTTTCCCTGTGAAGTGAA
TGAGCTGGAGCGGGATCGTGGTA	CAACTC	ACTATAGGGTTTTCCCTGTGAAGTGAA
TGAGCTGGAGCGGGATCGTGGTA	AGACTC	ACTATAGGGTTTTCCCTGTGAAGTGAA
TGAGCTGGAGCGGGATCGTGGTA	GAACTC	ACTATAGGGTTTTCCCTGTGAAGTGAA
TGAGCTGGAGCGGGATCGTGGTA	CGCCTC	ACTATAGGGTTTTCCCTGTGAAGTGAA
TGAGCTGGAGCGGGATCGTGGTA	GATCTC	ACTATAGGGTTTTCCCTGTGAAGTGAA
TGAGCTGGAGCGGGATCGTGGTA	AGTCTC	ACTATAGGGTTTTCCCTGTGAAGTGAA
TGAGCTGGAGCGGGATCGTGGTA	AGTATC	ACTATAGGGTTTTCCCTGTGAAGTGAA
TGAGCTGGAGCGGGATCGTGGTA	CAACTC	ACTATAGGGTTTTCCCTGTGAAGTGAA
TGAGCTGGAGCGGGATCGTGGTA	CGACTC	ACTATAGGGTTTTCCCTGTGAAGTGAA
TGAGCTGGAGCGGGATCGTGGTA	GGCCTC	ACTATAGGGTTTTCCCTGTGAAGTGAA
TGAGCTGGAGCGGGATCGTGGTA	CATCAG	ACTATAGGGTTTTCCCTGTGAAGTGAA
TGAGCTGGAGCGGGATCGTGGTA	GAACTC	ACTATAGGGTTTTCCCTGTGAAGTGAA
TGAGCTGGAGCGGGATCGTGGTA	CGTCAG	ACTATAGGGTTTTCCCTGTGAAGTGAA
TGAGCTGGAGCGGGATCGTGGTA	GGACTC	ACTATAGGGTTTTCCCTGTGAAGTGAA
TGAGCTGGAGCGGGATCGTGGTA	CGCCTC	ACTATAGGGTTTTCCCTGTGAAGTGAA
TGAGCTGGAGCGGGATCGTGGTA	GGCCTC	ACTATAGGGTTTTCCCTGTGAAGTGAA
TGAGCTGGAGCGGGATCGTGGTA	CAACTC	ACTATAGGGTTTTCCCTGTGAAGTGAA
TGAGCTGGAGCGGGATCGTGGTA	AGACTC	ACTATAGGGTTTTCCCTGTGAAGTGAA
TGAGCTGGAGCGGGATCGTGGTA	CAACTC	ACTATAGGGTTTTCCCTGTGAAGTGAA
TGAGCTGGAGCGGGATCGTGGTA	CAACTC	ACTATAGGGTTTTCCCTGTGAAGTGAA
TGAGCTGGAGCGGGATCGTGGTA	CGCTCG	ACTATAGGGTTTTCCCTGTGAAGTGAA
TGAGCTGGAGCGGGATCGTGGTA	GGTCTC	ACTATAGGGTTTTCCCTGTGAAGTGAA
TGAGCTGGAGCGGGATCGTGGTA	GGACTC	ACTATAGGGTTTTCCCTGTGAAGTGAA
TGAGCTGGAGCGGGATCGTGGTA	CAACTC	ACTATAGGGTTTTCCCTGTGAAGTGAA
TGAGCTGGAGCGGGATCGTGGTA	GCCCGA	ACTATAGGGTTTTCCCTGTGAAGTGAA
TGAGCTGGAGCGGGATCGTGGTA	TGACTC	ACTATAGGGTTTTCCCTGTGAAGTGAA
TGAGCTGGAGCGGGATCGTGGTA	CAACTC	ACTATAGGGTTTTCCCTGTGAAGTGAA
TGAGCTGGAGCGGGATCGTGGTA	CAACTC	ACTATAGGGTTTTCCCTGTGAAGTGAA
TGAGCTGGAGCGGGATCGTGGTA	GAACTC	ACTATAGGGTTTTCCCTGTGAAGTGAA
TGAGCTGGAGCGGGATCGTGGTA	GAACTC	ACTATAGGGTTTTCCCTGTGAAGTGAA
TGAGCTGGAGCGGGATCGTGGTA	CAACTC	ACTATAGGGTTTTCCCTGTGAAGTGAA
TGAGCTGGAGCGGGATCGTGGTA	GAACTC	ACTATAGGGTTTTCCCTGTGAAGTGAA
TGAGCTGGAGCGGGATCGTGGTA	GGGCTC	ACTATAGGGTTTTCCCTGTGAAGTGAA
TGAGCTGGAGCGGGATCGTGGTA	TGCCTC	ACTATAGGGTTTTCCCTGTGAAGTGAA
TGAGCTGGAGCGGGATCGTGGTA	AAACTC	ACTATAGGGTTTTCCCTGTGAAGTGAA
TGAGCTGGAGCGGGATCGTGGTA	GAACTC	ACTATAGGGTTTTCCCTGTGAAGTGAA
TGAGCTGGAGCGGGATCGTGGTA	CGACTC	ACTATAGGGTTTTCCCTGTGAAGTGAA
TGAGCTGGAGCGGGATCGTGGTA	CAACTC	ACTATAGGGTTTTCCCTGTGAAGTGAA
TAATA	CGACTC	ACTATA: Wild-type T7 promoter

a) The sequences are represented as 5'-to-3' orientation. The six nucleotides between spaces indicate the randomized region (positions -12 to -7). For reference, the wild-type T7 promoter sequence is shown at the bottom of the table.

Supplemental Table S4. Sequences of clones from template DNA pool for Syn5 promoter analysis[a]

GTTGAAGGCGGTGGAGGCGGATCGATTG	TGTGTC	CGTAA*GAGGAAAACCTCTTACG*	*GACACA*	*CACCAGAGACACGAGAACCAGCA*
GTTGAAGGCGGTGGAGGCGGATCGATTG	AGGGGT	CGTAA*GAGGAAAACCTCTTACG*	*ACCCCT*	*CACCAGAGACACGAGAACCAGCA*
GTTGAAGGCGGTGGAGGCGGATCGATTG	GCCTTG	CGTAA*GAGGAAAACCTCTTACG*	*CAAGGC*	*CACCAGAGACACGAGAACCAGCA*
GTTGAAGGCGGTGGAGGCGGATCGATTG	GACACC	CGTAA*GAGGAAAACCTCTTACG*	*GGTGTC*	*CACCAGAGACACGAGAACCAGCA*
GTTGAAGGCGGTGGAGGCGGATCGATTG	GCTGGC	CGTAA*GAGGAAAACCTCTTACG*	*GCCAGC*	*CACCAGAGACACGAGAACCAGCA*
GTTGAAGGCGGTGGAGGCGGATCGATTG	GTGTAG	CGTAA*GAGGAAAACCTCTTACG*	*CTACAC*	*CACCAGAGACACGAGAACCAGCA*
GTTGAAGGCGGTGGAGGCGGATCGATTG	GGTCAG	CGTAA*GAGGAAAACCTCTTACG*	*CTGACC*	*CACCAGAGACACGAGAACCAGCA*
GTTGAAGGCGGTGGAGGCGGATCGATTG	TGTTAT	CGTAA*GAGGAAAACCTCTTACG*	*ATAACA*	*CACCAGAGACACGAGAACCAGCA*
GTTGAAGGCGGTGGAGGCGGATCGATTG	GGCTCC	CGTAA*GAGGAAAACCTCTTACG*	*GGAGCC*	*CACCAGAGACACGAGAACCAGCA*
GTTGAAGGCGGTGGAGGCGGATCGATTG	TGTTGA	CGTAA*GAGGAAAACCTCTTACG*	*TCAACA*	*CACCAGAGACACGAGAACCAGCA*
GTTGAAGGCGGTGGAGGCGGATCGATTG	GGCCCT	CGTAA*GAGGAAAACCTCTTACG*	*AGGGCC*	*CACCAGAGACACGAGAACCAGCA*
GTTGAAGGCGGTGGAGGCGGATCGATTG	TCACAC	CGTAA*GAGGAAAACCTCTTACG*	*GTGTGA*	*CACCAGAGACACGAGAACCAGCA*
GTTGAAGGCGGTGGAGGCGGATCGATTG	CTTCTC	CGTAA*GAGGAAAACCTCTTACG*	*GAGAAG*	*CACCAGAGACACGAGAACCAGCA*
GTTGAAGGCGGTGGAGGCGGATCGATTG	TCGGGA	CGTAA*GAGGAAAACCTCTTACG*	*TCCCGA*	*CACCAGAGACACGAGAACCAGCA*
GTTGAAGGCGGTGGAGGCGGATCGATTG	CATTTC	CGTAA*GAGGAAAACCTCTTACG*	*GAAATG*	*CACCAGAGACACGAGAACCAGCA*
GTTGAAGGCGGTGGAGGCGGATCGATTG	AACTAC	CGTAA*GAGGAAAACCTCTTACG*	*GTAGTT*	*CACCAGAGACACGAGAACCAGCA*
GTTGAAGGCGGTGGAGGCGGATCGATTG	TGCCAT	CGTAA*GAGGAAAACCTCTTACG*	*ATGGCA*	*CACCAGAGACACGAGAACCAGCA*
GTTGAAGGCGGTGGAGGCGGATCGATTG	GCTCCA	CGTAA*GAGGAAAACCTCTTACG*	*TGGAGC*	*CACCAGAGACACGAGAACCAGCA*
GTTGAAGGCGGTGGAGGCGGATCGATTG	CTTGTA	CGTAA*GAGGAAAACCTCTTACG*	*TACAAG*	*CACCAGAGACACGAGAACCAGCA*
GTTGAAGGCGGTGGAGGCGGATCGATTG	CCTCGC	CGTAA*GAGGAAAACCTCTTACG*	*GCGAGG*	*CACCAGAGACACGAGAACCAGCA*
GTTGAAGGCGGTGGAGGCGGATCGATTG	CCGTAT	CGTAA*GAGGAAAACCTCTTACG*	*ATACGG*	*CACCAGAGACACGAGAACCAGCA*
GTTGAAGGCGGTGGAGGCGGATCGATTG	TGTCAC	CGTAA*GAGGAAAACCTCTTACG*	*GTGACA*	*CACCAGAGACACGAGAACCAGCA*
GTTGAAGGCGGTGGAGGCGGATCGATTG	GTCCCA	CGTAA*GAGGAAAACCTCTTACG*	*TGGGAC*	*CACCAGAGACACGAGAACCAGCA*
GTTGAAGGCGGTGGAGGCGGATCGATTG	TTCTAA	CGTAA*GAGGAAAACCTCTTACG*	*TTAGAA*	*CACCAGAGACACGAGAACCAGCA*
Wild-type Syn5 promoter:ATTG	GGCACC	CGTAA		

a) The sequences are represented as 5'-to-3' orientation. The six nucleotides between spaces indicate the randomized region. The transcribed region is emphasized by Italics. For reference, the wild-type Syn5 promoter sequence is shown at the bottom of the table.

Supplemental Table S5. Sequences of clones from the control Syn5 promoter pool[a]

GCTGGTTCTCGTGTCTCTGGTG	CCGGAT	CGTAAGAGGAAAACCTCGGC
GCTGGTTCTCGTGTCTCTGGTG	CCTGTG	CGTAAGAGGAAAACCTCGGC
GCTGGTTCTCGTGTCTCTGGTG	ACCTTT	CGTAAGAGGAAAACCTCGGC
GCTGGTTCTCGTGTCTCTGGTG	GTGGAT	CGTAAGAGGAAAACCTCGGC
GCTGGTTCTCGTGTCTCTGGTG	TGGGAT	CGTAAGAGGAAAACCTCGGC
GCTGGTTCTCGTGTCTCTGGTG	TGTCTA	CGTAAGAGGAAAACCTCGGC
GCTGGTTCTCGTGTCTCTGGTG	GCGGAT	CGTAAGAGGAAAACCTCGGC
GCTGGTTCTCGTGTCTCTGGTG	ATGGAT	CGTAAGAGGAAAACCTCGGC
GCTGGTTCTCGTGTCTCTGGTG	GTTAAA	CGTAAGAGGAAAACCTCGGC
GCTGGTTCTCGTGTCTCTGGTG	CCCGGG	CGTAAGAGGAAAACCTCGGC
GCTGGTTCTCGTGTCTCTGGTG	GTGGAT	CGTAAGAGGAAAACCTCGGC
GCTGGTTCTCGTGTCTCTGGTG	TTGTCG	CGTAAGAGGAAAACCTCGGC
GCTGGTTCTCGTGTCTCTGGTG	TTCTTA	CGTAAGAGGAAAACCTCGGC
GCTGGTTCTCGTGTCTCTGGTG	TCCGTC	CGTAAGAGGAAAACCTCGGC
GCTGGTTCTCGTGTCTCTGGTG	GGCCAA	CGTAAGAGGAAAACCTCGGC
GCTGGTTCTCGTGTCTCTGGTG	GTTCAA	CGTAAGAGGAAAACCTCGGC
GCTGGTTCTCGTGTCTCTGGTG	CTCTGG	CGTAAGAGGAAAACCTCGGC
GCTGGTTCTCGTGTCTCTGGTG	CATTTA	CGTAAGAGGAAAACCTCGGC
GCTGGTTCTCGTGTCTCTGGTG	CCGCAC	CGTAAGAGGAAAACCTCGGC
GCTGGTTCTCGTGTCTCTGGTG	TCCTCA	CGTAAGAGGAAAACCTCGGC
GCTGGTTCTCGTGTCTCTGGTT	GTCGGG	CGTAAGAGGAAAACCTCGGC
GCTGGTTCTCGTGTCTCTGGTG	CTTTCA	CGTAAGAGGAAAACCTCGGC
GCTGGTTCTCGTGTCTCTGGTG	TGGGAT	CGTAAGAGGAAAACCTCGGC
GCTGGTTCTCGTGTCTCTGGTG	GGAACG	CGTAAGAGGAAAACCTCGGC
GCTGGTTCTCGTGTCTCTGGTG	GAGGAT	CGTAAGAGGAAAACCTCGGC
GCTGGTTCTCGTGTCTCTGGTG	CTTAAC	CGTAAGAGGAAAACCTCGGC
GCTGGTTCTCGTGTCTCTGGTG	GTGGAT	CGTAAGAGGAAAACCTCGGC
GCTGGTTCTCGTGTCTCTGGTG	ATGGAT	CGTAAGAGGAAAACCTCGGC
GCTGGTTCTCGTGTCTCTGGTG	CCCCCT	CGTAAGAGGAAAACCTCGGC
GCTGGTTCTCGTGTCTCTGGTG	CTTCTC	CGTAAGAGGAAAACCTCGGC
GCTGGTTCTCGTGTCTCTGGTG	CTCTTG	CGTAAGAGGAAAACCTCGGC
GCTGGTTCTCGTGTCTCTGGTG	TCACTG	CGTAAGAGGAAAACCTCGGC
GCTGGTTCTCGTGTCTCTGGTG	TGTACT	CGTAAGAGGAAAACCTCGGC
GCTGGTTCTCGTGTCTCTGGTG	CTCTGC	CGTAAGAGGAAAACCTCGGC
GCTGGTTCTCGTGTCTCTGGTG	TGTTGG	CGTAAGAGGAAAACCTCGGC
GCTGGTTCTCGTGTCTCTGGTG	GTGCTG	CGTAAGAGGAAAACCTCGGC
GCTGGTTCTCGTGTCTCTGGTG	TATTTA	CGTAAGAGGAAAACCTCGGC
GCTGGTTCTCGTGTCTCTGGTG	GTGGAT	CGTAAGAGGAAAACCTCGGC
GCTGGTTCTCGTGTCTCTGGTG	TGCCTG	CGTAAGAGGAAAACCTCGGC
GCTGGTTCTCGTGTCTCTGGTG	GAGGTT	CGTAAGAGGAAAACCTCGGC
GCTGGTTCTCGTGTCTCTGGTG	GCTTTG	CGTAAGAGGAAAACCTCGGC
GCTGGTTCTCGTGTCTCTGGTG	CCGGAT	CGTAAGAGGAAAACCTCGGC
GCTGGTTCTCGTGTCTCTGGTG	AATTGA	CGTAAGAGGAAAACCTCGGC
GCTGGTTCTCGTGTCTCTGGTG	TATACT	CGTAAGAGGAAAACCTCGGC
GCTGGTTCTCGTGTCTCTGGTG	GTATCA	CGTAAGAGGAAAACCTCGGC
GCTGGTTCTCGTGTCTCTGGTG	TTCGCT	CGTAAGAGGAAAACCTCGGC
GCTGGTTCTCGTGTCTCTGGTG	GGCATA	CGTAAGAGGAAAACCTCGGC
GCTGGTTCTCGTGTCTCTGGTG	ACGGCA	CGTAAGAGGAAAACCTCGGC
GCTGGTTCTCGTGTCTCTGGTG	TCCCGC	CGTAAGAGGAAAACCTCGGC
GCTGGTTCTCGTGTCTCTGGTG	GCATTT	CGTAAGAGGAAAACCTCGGC
GCTGGTTCTCGTGTCTCTGGTG	ACTCCA	CGTAAGAGGAAAACCTCGGC
GCTGGTTCTCGTGTCTCTGGTG	AGTTAC	CGTAAGAGGAAAACCTCGGC
GCTGGTTCTCGTGTCTCTGGTG	GAGGTG	CGTAAGAGGAAAACCTCGGC
GCTGGTTCTCGTGTCTCTGGTG	GTCGAC	CGTAAGAGGAAAACCTCGGC
GCTGGTTCTCGTGTCTCTGGTG	CTGGAT	CGTAAGAGGAAAACCTCGGC
GCTGGTTCTCGTGTCTCTGGTG	TGTTCT	CGTAAGAGGAAAACCTCGGC
GCTGGTTCTCGTGTCTCTGGTG	TTCACC	CGTAAGAGGAAAACCTCGGC
ATTG	GGCACC	CGTAA: Wild-type Syn5 promoter

a) The sequences are represented as 5'-to-3' orientation. The six nucleotides between spaces indicate the randomized region (positions -11 to -6). For reference, the wild-type Syn5 promoter sequence is shown at the bottom of the table.

Supplemental Table S6. Sequences of clones from the enriched Syn5 promoter pool[a]

GCTGGTTCTCGTGTCTCTGGTG	GACGCC	CGTAAGAGGAAAACCTCGGC
GCTGGTTCTCGTGTCTCTGGTG	GGTCCT	CGTAAGAGGAAAACCTCGGC
GCTGGTTCTCGTGTCTCTGGTG	GTCACC	CGTAAGAGGAAAACCTCGGC
GCTGGTTCTCGTGTCTCTGGTG	ATTGTG	CGTAAGAGGAAAACCTCGGC
GCTGGTTCTCGTGTCTCTGGTG	GGTCCG	CGTAAGAGGAAAACCTCGGC
GCTGGTTCTCGTGTCTCTGGTG	ATGTAT	CGTAAGAGGAAAACCTCGGC
GCTGGTTCTCGTGTCTCTGGTG	GGTTCT	CGTAAGAGGAAAACCTCGGC
GCTGGTTCTCGTGTCTCTGGTG	GGTTCA	CGTAAGAGGAAAACCTCGGC
GCTGGTTCTCGTGTCTCTGGTG	GGCTCG	CGTAAGAGGAAAACCTCGGC
GCTGGTTCTCGTGTCTCTGGTG	GGCCTC	CGTAAGAGGAAAACCTCGGC
GCTGGTTCTCGTGTCTCTGGTG	GCAGCC	CGTAAGAGGAAAACCTCGGC
GCTGGTTCTCGTGTCTCTGGTG	GATGGG	CGTAAGAGGAAAACCTCGGC
GCTGGTTCTCGTGTCTCTGGTG	GCTGCC	CGTAAGAGGAAAACCTCGGC
GCTGGTTCTCGTGTCTCTGGTG	GTCACT	CGTAAGAGGAAAACCTCGGC
GCTGGTTCTCGTGTCTCTGGTG	GCCCGT	CGTAAGAGGAAAACCTCGGC
GCTGGTTCTCGTGTCTCTGGTG	GTCACC	CGTAAGAGGAAAACCTCGGC
GCTGGTTCTCGTGTCTCTGGTG	GGTACC	CGTAAGAGGAAAACCTCGGC
GCTGGTTCTCGTGTCTCTGGTG	GGTCTG	CGTAAGAGGAAAACCTCGGC
GCTGGTTCTCGTGTCTCTGGTG	GGCACG	CGTAAGAGGAAAACCTCGGC
GCTGGTTCTCGTGTCTCTGGTG	GGCTCC	CGTAAGAGGAAAACCTCGGC
GCTGGTTCTCGTGTCTCTGGTG	GGTCGA	CGTAAGAGGAAAACCTCGGC
GCTGGTTCTCGTGTCTCTGGTG	GCCCCG	CGTAAGAGGAAAACCTCGGC
GCTGGTTCTCGTGTCTCTGGTG	TGGGAT	CGTAAGAGGAAAACCTCGGC
GCTGGTTCTCGTGTCTCTGGTG	GTCACC	CGTAAGAGGAAAACCTCGGC
GCTGGTTCTCGTGTCTCTGGTG	GTCATC	CGTAAGAGGAAAACCTCGGC
GCTGGTTCTCGTGTCTCTGGTG	GACACT	CGTAAGAGGAAAACCTCGGC
GCTGGTTCTCGTGTCTCTGGTG	GGTACA	CGTAAGAGGAAAACCTCGGC
GCTGGTTCTCGTGTCTCTGGTG	GGCGCG	CGTAAGAGGAAAACCTCGGC
GCTGGTTCTCGTGTCTCTGGTG	GGCTCC	CGTAAGAGGAAAACCTCGGC
GCTGGTTCTCGTGTCTCTGGTG	GTGCCG	CGTAAGAGGAAAACCTCGGC
GCTGGTTCTCGTGTCTCTGGTG	GACCCA	CGTAAGAGGAAAACCTCGGC
GCTGGTTCTCGTGTCTCTGGTG	GATTTA	CGTAAGAGGAAAACCTCGGC
GCTGGTTCTCGTGTCTCTGGTG	AGGAGC	CGTAAGAGGAAAACCTCGGC
GCTGGTTCTCGTGTCTCTGGTG	GGCGCA	CGTAAGAGGAAAACCTCGGC
GCTGGTTCTCGTGTCTCTGGTG	TTTCGA	CGTAAGAGGAAAACCTCGGC
GCTGGTTCTCGTGTCTCTGGTG	GATCCA	CGTAAGAGGAAAACCTCGGC
GCTGGTTCTCGTGTCTCTGGTG	GGGCCG	CGTAAGAGGAAAACCTCGGC
GCTGGTTCTCGTGTCTCTGGTG	GGCGCC	CGTAAGAGGAAAACCTCGGC
GCTGGTTCTCGTGTCTCTGGTG	GGTCTT	CGTAAGAGGAAAACCTCGGC
GCTGGTTCTCGTGTCTCTGGTG	TAGGAG	CGTAAGAGGAAAACCTCGGC
GCTGGTTCTCGTGTCTCTGGTG	GTTCAA	CGTAAGAGGAAAACCTCGGC
GCTGGTTCTCGTGTCTCTGGTG	GGCCTA	CGTAAGAGGAAAACCTCGGC
GCTGGTTCTCGTGTCTCTGGTG	GCTCCC	CGTAAGAGGAAAACCTCGGC
GCTGGTTCTCGTGTCTCTGGTG	GACTCC	CGTAAGAGGAAAACCTCGGC
GCTGGTTCTCGTGTCTCTGGTG	GATACG	CGTAAGAGGAAAACCTCGGC
GCTGGTTCTCGTGTCTCTGGTG	GGTCTG	CGTAAGAGGAAAACCTCGGC
GCTGGTTCTCGTGTCTCTGGTG	GGCTCG	CGTAAGAGGAAAACCTCGGC
GCTGGTTCTCGTGTCTCTGGTG	GTGCGT	CGTAAGAGGAAAACCTCGGC
GCTGGTTCTCGTGTCTCTGGTG	GGCCGA	CGTAAGAGGAAAACCTCGGC
GCTGGTTCTCGTGTCTCTGGTG	TGTCCA	CGTAAGAGGAAAACCTCGGC
ATTG	GGCACC	CGTAA: Wild-type Syn5 promoter

a) The sequences are represented as 5'-to-3' orientation. The six nucleotides between spaces indicate the randomized region (positions -11 to -6). For reference, the wild-type Syn5 promoter sequence is shown at the bottom of the table.
